# Unilateral craniosynostosis associated with ZIC1 gene mutation: a case report

**DOI:** 10.1093/jscr/rjaf1065

**Published:** 2026-01-08

**Authors:** Fahad K Alsharef, Khulood K Alraddadi, Tariq Aljared

**Affiliations:** College of Medicine, King Saud bin Abdulaziz University for Health Sciences (KSAU-HS), Sheikh Jaber Al-Sabah Road, Khashm Al An District, PO Box 3660, Riyadh 11481, Riyadh Province, Saudi Arabia; Department of Pediatric Surgery, Division of Pediatric Neurosurgery, King Abdullah Specialized Children Hospital, Ministry of National Guard Health Affairs, Ar Rimayah District, PO Box 22490, Riyadh 11426, Riyadh Province, Saudi Arabia; College of Medicine, King Saud bin Abdulaziz University for Health Sciences (KSAU-HS), Sheikh Jaber Al-Sabah Road, Khashm Al An District, PO Box 3660, Riyadh 11481, Riyadh Province, Saudi Arabia; Department of Pediatric Surgery, Division of Pediatric Neurosurgery, King Abdullah Specialized Children Hospital, Ministry of National Guard Health Affairs, Ar Rimayah District, PO Box 22490, Riyadh 11426, Riyadh Province, Saudi Arabia

**Keywords:** craniosynostosis, coronal, ZIC1, genetic, neurodevelopmental

## Abstract

Craniosynostosis is one of the most common craniofacial anomalies, resulting from premature fusion of one or more cranial sutures. While most cases are sporadic, a significant number have a genetic etiology, including monogenic mutations. Coronal synostosis, in particular, is frequently associated with genetic variants. Mutations in the zinc finger protein of cerebellum 1 (ZIC1) gene have recently been recognized as a rare cause of coronal craniosynostosis. We report an 11-month-old female infant with a ZIC1 mutation presenting with unilateral left coronal craniosynostosis, microcephaly, and multiple neurodevelopmental and systemic comorbidities. Due to progressive deformity and concerns of raised intracranial pressure, anterior cranial vault expansion with fronto-orbital advancement was performed, resulting in immediate cosmetic improvement. The postoperative course was uneventful, and developmental progress was noted on follow-up. This case illustrates an uncommon presentation within the ZIC1 associated craniosynostosis spectrum and highlights the importance of considering ZIC1 mutations in unexplained unilateral coronal cases, guiding genetic counseling, and surveillance.

## Introduction

Craniosynostosis is a congenital premature fusion of one or more of the skull sutures, leading to an abnormal skull morphology and craniofacial asymmetry [1]. It is the second most common craniofacial anomaly with a prevalence of 1 in 2250 births [2, 3]. Craniosynostosis is non-syndromic in most cases (85%), or a part of the 180 known syndromes associated with genetic mutations (15%). Familial cases constitute 8% of all cases, whereas the rest are sporadic [4]. Almost 80% of cases involve one suture, in contrast to syndromic types where more sutures are likely involved [1]. Among sutures involved, 1 in 10 000 children have coronal sutures fusion either unilateral or bilateral [2]. Monogenic etiologies are mostly detected in patients exhibiting coronal synostosis [5]. Genetic testing reveals a specific mutation in about 60% of bicoronal and 30% of unicoronal craniosynostosis even without defined syndromes [5, 6]. In addition to established genes, zinc finger protein of cerebellum 1 (ZIC1) [MIM:600470], located on chromosome 3q25.1, has been implicated in recent years as an additional genetic factor in the development of coronal craniosynostosis [7]. Herein, we report a case of 11-month-old female infant presenting with a rare ZIC1 mutation associated with plagiocephaly and multiple comorbidities.

## Case presentation

**Figure 1:**
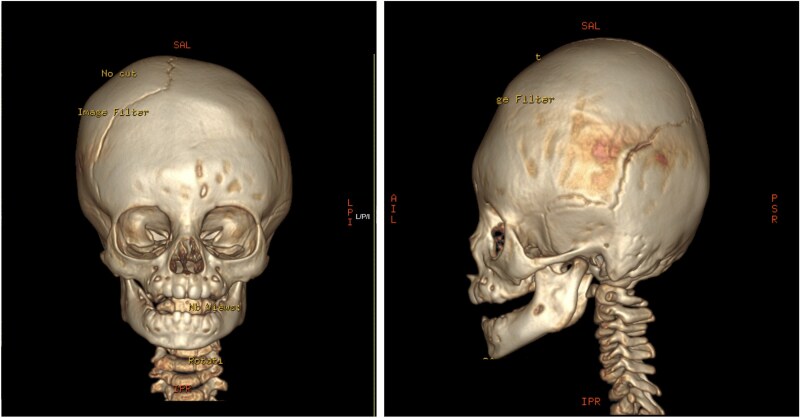
Pre-operative 3D reconstruction CT showing left plagiocephaly with towering, and a subtle harlequin sign.

**Figure 2:**
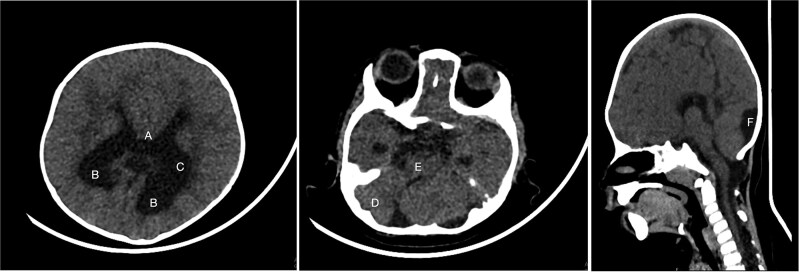
Brain CT showing: A-corpus callosum agenesis, B-colpocephaly, C-mild ventriculomegaly, D-right cerebellar and E-pontine hypoplasia, F-large cisterna magna.

**Figure 3:**
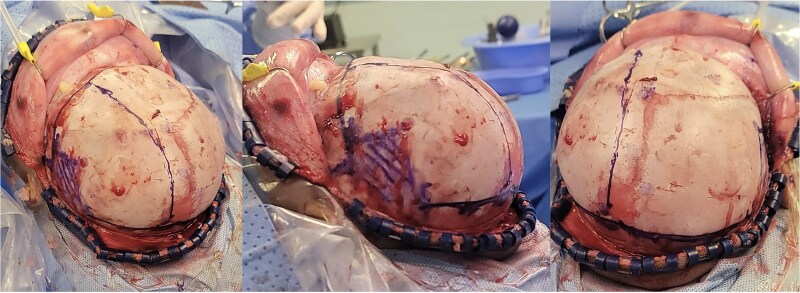
Intra-operative gross appearance of the left unilateral plagiocephaly with towering.

**Figure 4:**
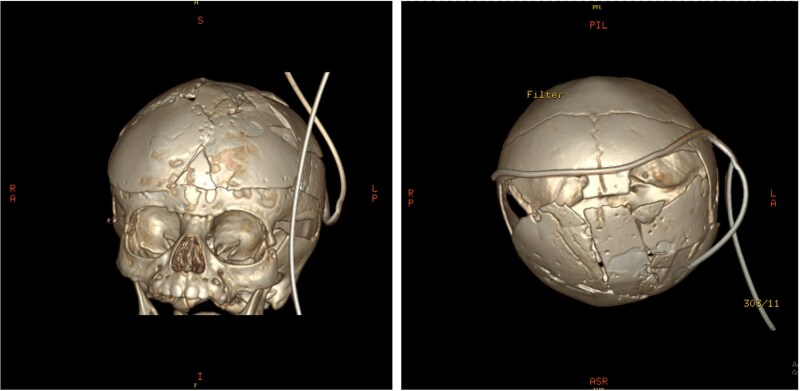
Post-operative 3D reconstruction CT showing skull shape correction and cosmetic results.

**Figure 5:**
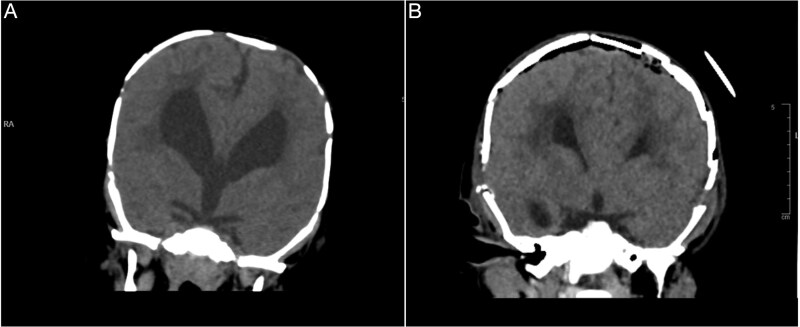
Brain CT bone window illustrating ventriculomegaly and bone resorption at six-month follow-up (A), compared to immediate post operative image (B).

**Figure 6:**
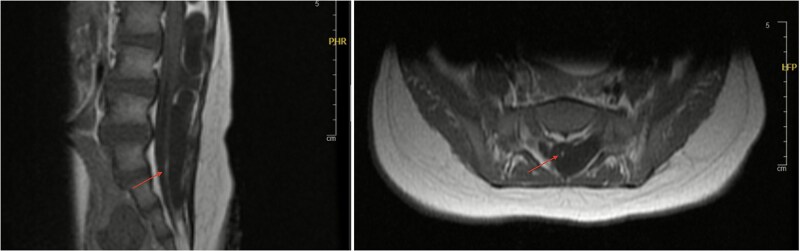
Axial and sagittal views of spinal MRI showing tethering of the cord at the level of S1 (arrows).

An 11-month-old female infant, born to unrelated Saudi parents, was referred to our facility with unilateral left coronal craniosynostosis, congenital microcephaly, global developmental delay, and a confirmed ZIC1 gene mutation. Comorbidities included a patent foramen ovale, aortopulmonary collateral vessel, G6PD deficiency, and gastroesophageal reflux disease (GERD). At birth head circumference was 29 cm (<first percentile), and progressive skull asymmetry and squinting were noted over time. Developmentally, she exhibited delayed gross and fine motor skills with limited object transfer. On examination, she was alert and visually tracking, with inward ocular deviation (esotropia). Head circumference was 37 cm (<first percentile) with anterior plagiocephaly and towering of the left frontal region (Fig. 1). Pupils were equal and reactive. Fundoscopy and cranial nerve examinations were unremarkable. Gross motor power was within normal limits, except for moderate lower limb spasticity. 3D reconstruction computed tomography (CT) confirmed premature fusion of the left coronal suture with subtle elevation of the superolateral orbital rim, indicating harlequin sign (Fig. 1). Brain CT showed corpus callosum agenesis, colpocephaly, mild ventriculomegaly, right cerebellar and pontine hypoplasia, and a large cisterna magna, consistent with the reported ZIC1-related malformation spectrum (Fig. 2). Given the progressive deformity and concerns of increased intracranial pressure that might be partially contributing to her neurodevelopmental delay, anterior cranial vault expansion with fronto-orbital advancement was performed jointly by the neurosurgery and plastic craniofacial teams (Fig. 3). Intraoperatively, left-sided dural tension was appreciated, indicating localized increased intracranial pressure. The orbital bandeau was reshaped and advanced, achieving immediate cosmetic improvement (Fig. 4). The patient tolerated the surgery well and recovered uneventfully. At her 4-month follow-up, she showed developmental progress including standing without support, purposeful hand use, and verbalization (“Baba”). At 6-month follow-up, brain CT revealed areas of bone resorption which led to the placement of a ventriculo-peritoneal shunt (Fig. 5). The known association of ZIC1 mutation with tethering of the cord prompted spinal magnetic resonance imaging (MRI) screening. Positive findings indicated an untethering procedure, which was successfully done (Fig. 6).

## Discussion

Craniosynostosis is a clinically and genetically heterogenous disorder, with chromosomal and single gene abnormalities accounting for almost on fifth of cases. Coronal suture involvement is the most linked subtype to an identifiable molecular basis [5]. Among the less common but increasingly recognized contributors is the ZIC1 gene, a transcription factor essential for neural crest differentiation, suture mesenchyme maintenance, and cerebellar development [7–9]. ZIC1 regulates developmental pathways, including Wnt, bone morphogenetic protein (BMP), Hedgehog signaling, and controls downstream targets such as EN2 (engrailed-2) that are essential for appropriate coronal suture positioning [7, 9]. Functional studies demonstrate that ZIC1 gain-of-function mutations enhance EN2 expression, promoting premature coronal fusion, whereas loss-of-function variants cause abnormal mineralization, ectopic suture formation, and neurodevelopmental defects [7–10]. These mechanisms explain pathogenic variants’ selectivity in predisposing to coronal craniosynostosis. Clinical manifestations of ZIC1 mutations extend beyond isolated suture fusion to include microcephaly, corpus callosum agenesis, cerebellar dysplasia, ventriculomegaly, tethered cord, scoliosis, and global developmental delay [10]. Our patient aligns with this broader phenotype, exhibiting most of these manifestations. These findings support that ZIC1 related craniosynostosis often occurs as a part of a broader neurodevelopmental phenotype rather than an isolated cranial abnormality. Most reported ZIC1 mutation cases describe bicoronal synostosis [7]. Our patient presented with unilateral coronal involvement, highlighting the variable expressivity. To our knowledge, only one reported ZIC1 mutation case exhibited unilateral craniosynostosis in the literature [9]. Reviews of craniosynostosis genetics highlight that incomplete penetrance and laterality variability are features of transcription factor related synostosis, including ZIC1 [1, 11]. Recognizing that unilateral presentations can also be associated with genetic mutations is important, since genetic testing is usually prioritized for syndromic or bicoronal cases. Our case therefore supports extending ZIC1 gene testing in unexplained unilateral coronal synostosis. Standard genetic testing identifies fibroblast growth factor receptor 2 (FGFR2), fibroblast growth factor receptor 3 (FGFR3), and twist-related protein 1 (TWIST1) mutations, but next generation sequencing has extended the diagnostic yield, revealing infrequent contributors, including ZIC1 [11]. Establishing molecular diagnoses clarifies prognosis and guides monitoring of associated anomalies [1]. Identification of the ZIC1 mutation in our case explained both the craniosynostosis and neurodevelopmental features, facilitating clear counseling of expected outcomes and limitations. Surgical intervention remains the mainstay of treatment for single gene mutation craniosynostosis. Genetically determined coronal synostosis may require more extensive or repeat surgeries [5]. Although surgery cannot reverse underlying neurodevelopmental deficits, timely intervention may prevent secondary complications and can improve cranial shape and quality of life [5, 9].

## Conclusion

This case adds to the very limited literature, illustrating an uncommon presentation of ZIC1 associated unilateral coronal synostosis in an infant with multiple comorbidities. The patient’s favorable postoperative outcome highlights the importance of timely surgical intervention for its functional and cosmetic benefits, even though developmental delay is multifactorial. Our findings add to the clinical spectrum of ZIC1 mutations, supporting its inclusion in diagnostic gene panels, and highlighting the heterogeneity of genotype–phenotype correlations.
